# The Functions and Mechanisms of Action of Insulators in the Genomes of Higher Eukaryotes

**DOI:** 10.32607/actanaturae.11144

**Published:** 2020

**Authors:** L. S. Melnikova, P. G. Georgiev, A. K. Golovnin

**Affiliations:** Institute of Gene Biology, Russian Academy of Sciences, Moscow, 119334 Russia

**Keywords:** insulator proteins, enhancer-promoter communication, chromatin loops, regulation of transcription, Su(Hw), TAD

## Abstract

The mechanisms underlying long-range interactions between chromatin regions and the principles of chromosomal architecture formation are currently under extensive scrutiny. A special class of regulatory elements known as insulators is believed to be involved in the regulation of specific long-range interactions between enhancers and promoters. This review focuses on the insulators of *Drosophila *and mammals, and it also briefly characterizes the proteins responsible for their functional activity. It was initially believed that the main properties of insulators are blocking of enhancers and the formation of independent transcription domains. We present experimental data proving that the chromatin loops formed by insulators play only an auxiliary role in enhancer blocking. The review also discusses the mechanisms involved in the formation of topologically associating domains and their role in the formation of the chromosomal architecture and regulation of gene transcription.

## INTRODUCTION

In higher eukaryotic cells, transcription, one of the key stages of gene expression, results from the interaction between promoters that determine transcription initiation and its basic level and the various *cis*-regulatory elements that either amplify (enhancers) or weaken (silencers) the transcription [1, 2, 3]. Enhancers and silencers may reside at a considerable distance from the genes whose transcription they regulate and be separated from them by numerous “alien” genes with their own regulation systems [4, 5]. In order to explain the mechanism of specific interactions between an enhancer/silencer and a promoter, a model has been proposed postulating that chromosomes are subdivided into transcription (chromatin) domains that strictly limit contacts between regulatory genome sequences [6].

A new class of regulatory elements called insulators was found for the first time in studies conducted using the fruit fly, *Drosophila melanogaster *[7, 8, 9]. Initially, two of the properties of insulators were described. First, insulators residing between the enhancer and the promoter prevent their interaction (an enhancer-blocking activity). Second, insulators surrounding the transgene neutralize the negative or positive effect of the adjacent chromatin on its expression (a barrier activity). Insulators have been detected in the genomes of all well-studied higher eukaryotes [10, 11]. It was initially assumed that insulators that interact with each other are responsible for the formation of isolated transcription domains. However, further research has demonstrated that insulators are multifunctional elements comprised by the regulation systems of many genes [12-18].

## INSULATORS IN THE GENOMES OF HIGHER EUKARYOTES

The fruit fly *Drosophila melanogaster *was often used as a model organism in the first studies focused on insulators. By then, a system based on *P*-transposon enabling efficient transgenic modification of the fruit fly genome had already been developed [19]. It was not until much later that the methods for *in vivo *genome modification of vertebrate animals were developed [20, 21]. *P*-dependent integration has a stochastic nature, allowing one to study the effect of different chromosomal environments on transgenic expression. The *white *gene responsible for eye pigmentation in *Drosophila melanogaster *was often used as a reporter gene [22]. In different transgenic lines carrying the *white *gene without enhancers (*mini-white*), the eye color in flies ranged from pale yellow to red, being caused by transgene integration sites. This phenomenon is known as the chromosomal position effect [22, 23]. It was assumed that expression of the *mini-white *gene depends on the chromosomal position due to the activity of genome enhancers residing near the transgene integration site. However, it was proved later that in more than 70% of cases, the *mini-white *transcription initiated in the surrounding genome regions is responsible for the activating effect of the chromosomal environment [24].

The first insulators described in the *Drosophila melanogaster *genome were the *scs *and *scs’ *(specialized chromatin structure) sequences found at the cytogenetic locus 87A7 as nuclease-hypersensitive DNA regions surrounding a cluster of five genes, including two genes coding for heat shock proteins 70 (*hsp70*) [8, 9, 25]. Activation of the *hsp70 *genes induces decondensation of chromomer 87A7 to form a “puff” in salivary gland polytene chromosomes. Cytological studies showed that the *scs *and *scs’ *elements reside at sites where the decondensed 87A7 locus is flanked by condensed chromatin. However, it was revealed later that *scs *and *scs’ *are located inside the puff rather than at its boundaries and do not restrict the 87A7 decondensation [26]. It was suggested that *scs *and *scs’ *are the boundaries of the transcription domain that includes the *hsp70 *genes. The *scs *and *scs’ *elements within transgenes exhibited enhancer-blocking and barrier insulator properties [8, 9]. Next, it was shown that the *scs *(993 bp) and *scs’ *(500 bp) insulators have a complex structure that includes the gene promoters and transcription termination signals [27-30].

**Fig. 1:**
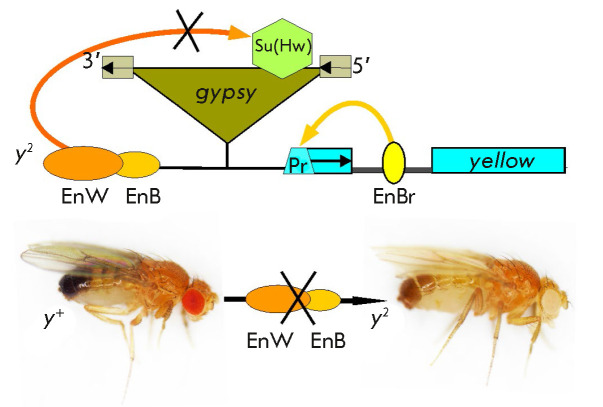
Schematic representation of the y2 allele. Exons of the *yellow *gene are shown with rectangles, with an arrow indicating the direction of transcription; EnW – wing enhancer; EnB – body enhancer; EnBr – bristle enhancer; and Pr – promoter of the gene. The *gypsy *retrotransposon is depicted as a triangle; the rectangles at its ends are long terminal repeats, with their direction shown with arrows. The Su(Hw) insulator is depicted as a hexagon inside *gypsy*. The photographs show the phenotypes of flies: *y+ *– wild type, the *yellow *gene is expressed in all cuticular structures; *y2 *– body and wing enhancers are blocked by the Su(Hw) insulator (depicted as strikethrough); the *yellow *gene is not expressed in the body cuticle and wings but continues to be expressed in the bristles

The best studied insulator of *Drosophila melanogaster *was found in the regulatory region of the *gypsy *retrotransposon (Mdg4) [31]. The *gypsy *retrotransposon affects the expression of the neighboring genes by causing mutant phenotypes. The effect of *gypsy *on transcription is due to a 460-bp sequence located in its 5’-transcribed untranslated region [7, 32]. In transgenic lines, the *gypsy *insulator blocks the activity of various enhancers at all stages of Drosophila development [33-36]. The insulator was found to consist of 12 degenerated octameric sites of Su(Hw) protein binding [32, 37, 38]. The properties of the *gypsy *insulator were initially tested using the regulatory system of the *yellow *locus responsible for the pigmentation of cuticle structures in embryos, larvae, and the imago of fruit flies [39]. Enhancers controlling the transcription of *yellow *in the wing plates and body cuticle reside in the 5’ gene region, while the enhancers controlling expression in bristles reside in the intron [7]. In the *y2 *allele, the *gypsy *retrotransposon is integrated in the 5’ region of the *yellow *gene, between the promoter and enhancers activating transcription in the wings and body. As a result, the insulator blocks the body and wing enhancers but does not affect the activity of the bristle enhancer residing in the gene intron (*Fig. 1*). A mutation inactivating the *su(Hw) *gene makes the insulator in the *y2 *allele disappear, thus completely restoring *yellow *gene expression [40]. Several studies have shown that when transgene is integrated into the heterochromatin regions of the genome or in the vicinity of the Polycomb response element (PRE)-dependent silencer, the *gypsy *insulator efficiently protects the *white *reporter gene against repression [41, 42].

Another insulator was found in the long terminal repeat of the *Idefix *retrotransposon [43]. The barrier activity of the *Idefix *insulator and its ability to block various enhancers were detected using transgenic lines [44].

The first functional genomic insulator, 1A2, containing two Su(Hw) protein binding sites was found in the 3’ region of the *yellow *gene [45, 46]. It turned out that many genome sequences, including 1–3 binding sites for Su(Hw), act as insulators in transgenes [47, 48, 49]. However, it was found using synthetic repetitive Su(Hw)-binding sites that at least four sites provide efficient insulator activity [50]. This contradiction can be attributed to the existence of proteins that have not been identified yet, which are involved in the formation of functional endogenous insulators, along with Su(Hw) [51].

The genome of *Drosophila melanogaster *was found to contain many insulator sequences not carrying binding sites for the Su(Hw) protein. These include the SF1 and SF2 insulators from the *Antennapedia *complex (ANT-C) [52, 53]; *facet-strawberry *sequences protecting the *Notch *gene against the effects of the surrounding chromatin [54]; the *Wari *insulator [55] residing at the 3’ end of the *white *gene; and the ME boundary element inhibiting the activity of the enhancer from the *eyeless *gene with respect to the promoter of the neighboring *myoglianin *gene [56]. The boundaries of independent transcription domains, *Mcp*, *Fab-6*, *Fab-7*, and *Fab-8, *demonstrating properties of the insulators in transgenic lines have been revealed in the regulatory region of the *Bithorax *complex (BX-C) [57-71].

The first vertebrate insulators were found at the boundaries of clusters of transcriptionally active genes and heterochromatin regions. The HS4 insulator was detected at the 5’ end of the chicken β-globin locus [72]. The core sequence of HS4 contains the CTCF-binding site [73]. Subsequently, searching for new vertebrate insulators was often based on testing DNA fragments containing CTCF-binding sites [74, 75]. Thus, an insulator containing four CTCF-binding sites and playing a crucial role in the imprinted expression of the *Igf2/H19 *locus was found in mice and humans [76, 77, 78]. Many CTCF-dependent vertebrate insulators have been described, being consistent with the views on the key role played by the CTCF protein in the organization of chromatin architecture [74, 75].

## THE MODELS OF THE MECHANISM OF ACTION OF INSULATORS

The data on the properties of insulators were used to propose two groups of alternative models for explaining their mechanism of action.

**Fig. 2:**
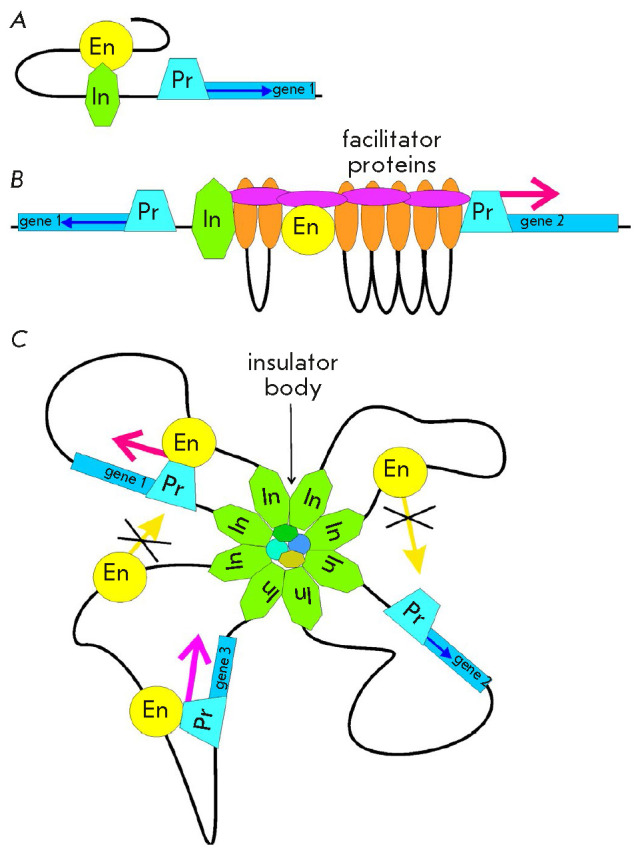
The models of insulator function. (*A*) Model of an “enhancer decoy.” (*B*) Blocking of facilitating proteins. (*C*) Structural model. Formation of independent transcriptional domains. Designations: En – enhancer; In – insulator; and Pr – promoter. Red arrows indicate transcription activation by a specific enhancer; blue arrows show the basic activity of the promoter. Strikethrough arrows indicate blocking interactions between enhancers and promoters from the adjacent domains

According to the transcription models, an insulator actively interrupts the specific long-range enhancer-promoter interactions [73, 79, 80]. Depending on the possible mechanism of enhancer-promotor interactions, different variants of insulator action were considered. According to one model, the enhancer “looks for” a promoter by moving along the chromatin fibril. In this case, the insulator acts as a physical barrier preventing enhancer motion. It was also supposed that insulators are pseudo-promoters. They do not initiate transcription but can interact with enhancers, thus inhibiting their activity (*Fig. 2a*). According to another popular model, long-range enhancer-promotor contacts are ensured by special facilitating proteins. For example, the mammalian homodimerizing protein LDB1 forms specific contacts between the enhancers and promoters of many genes [81]. The *Drosophila melanogaster *CHIP protein facilitates enhancer-promotor interactions in the *cut *locus [82]. The CHIP protein was shown to interact with the components of the *gypsy *insulator [83, 84]. When the enhancer-promotor interaction is weakened by a CHIP mutation, Su(Hw)-dependent insulation becomes more efficient. Hence, the insulator can inhibit the activity of the facilitating proteins that ensure the enhancer-promotor communication (*Fig. 2B*).

The structural models of the action of insulators have gained wide popularity [85]. Initially, these models were based on the idea that chromosomes form large independent chromatin loops [6]. It was assumed that chromatin loops are independent transcription domains and block any interactions between the regulatory elements in neighboring domains.

Later, studies focused on localizing the Su(Hw) protein on chromosomes and in the nucleus substantially gained in importance. It was believed that polytene chromosome bands correspond to transcription domains, while interbands correspond to their boundaries. It was shown that the binding sites of Su(Hw) reside in some interbands (i.e., limit the transcription domains) [86]. In Drosophila cultured cells, embryos, and imaginal discs, the Su(Hw) protein was found within compact nuclear structures known as insulator bodies [86]. It was assumed that each insulator body consists of multiple individual insulators that interact with each other and divide the chromatin fibril into domain loops, thus forming rosette-like structures (*Fig. 2C*). The insulators lying in the base of the rosette can interact with the nuclear lamina (shell) or with components of the nuclear pore, thus laying the basis for the spatial organization of chromatin. The structural models postulate that the key role of insulators is to form chromatin loops, while their activity is believed to result from this organization. Chromatin looping may either topologically or physically impede interaction between enhancers and promoters located in neighboring domains [87].

Today, the structural models rely on data on the organization of higher eukaryotic chromosomes into topologically associating domains (TADs) [88-91]. A hypothesis has been put forward that insulators are the TAD boundaries. The interaction between insulators gives rise to chromatin loops limiting the enhancer activity.

## Su(Hw)-DEPENDENT COMPLEX AS A MODEL FOR STUDYING INSULATORS

Insulator activity is ensured by a complex of interacting proteins that bind to the insulator DNA sequence. In many studies, the mechanisms of insulator functioning and formation in *Drosophila melanogaster *were investigated for the Su(Hw)-dependent complex.

**Fig. 3:**
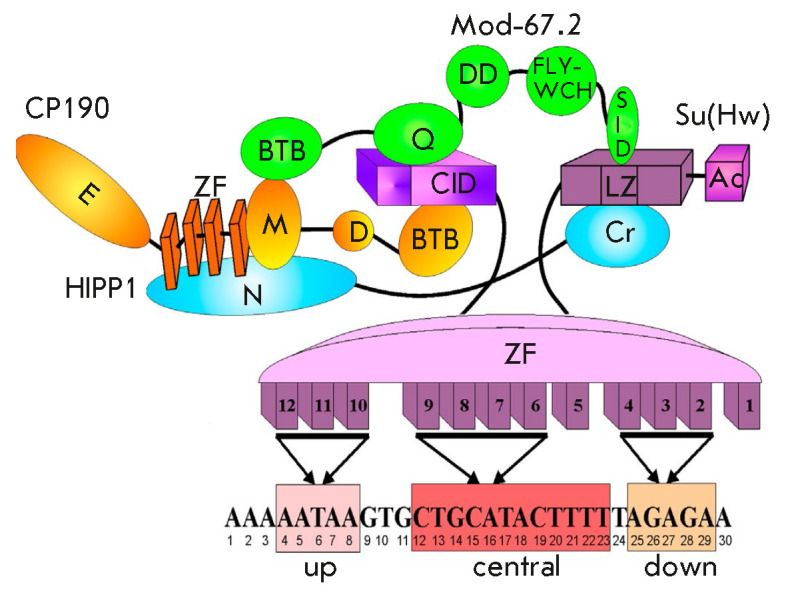
A model of Su(Hw)-dependent insulator complex formation. The domains of the Su(Hw) protein are shown in lilac; Mod(mdg4)-67.2 protein domains are shown in green; CP190 protein domains, in orange; and HIPP1 protein domains, in blue. Domain abbreviations: CID – CP190 interacting domain; Ac – C-terminal acidic domain; ZF – zinc finger domain; LZ – leucine zipper; BTB – BTB/POZ domain; Q – glutamine-rich region; DD – dimerization domain; FLYWCH – FLYWCH type zinc finger; SID – Su(Hw) interacting domain; D – asparagine- rich domain; M – the microtubule and centrosome associated domain; E – glutamine-rich C-terminal domain. Below is the consensus binding sequence for the Su(Hw) protein from the *gypsy *insulator. The ZFs binding each motif are shown with arrows

The key protein of the complex, Su(Hw), is expressed during the entire development process and is found in most *Drosophila melanogaster *tissues. Inactivation of the *su(Hw) *gene results in female sterility [35, 92]. The Su(Hw) protein consists of the N-terminal region rich in acidic amino acids, a DNA-binding domain containing twelve C2H2-type zinc fingers (ZFs), and the C-terminal region, which is also rich in acidic amino acid residues [92]. Su(Hw) binds to a consensus sequence (~ 26 bp) consisting of three modules [93]. Cluster ZF6-9 binds to the main (central) module; cluster ZF2-4, to the CG-rich module (“down”); and cluster ZF10-12, to the AT-rich module (“up”) (*Fig. 3*). The tenth ZF affects the efficiency of protein binding to some sites [93, 94]. For example, a mutation in ZF10 makes it impossible for the Su(Hw) protein to efficiently bind to the *gypsy *insulator sequence [51]. The C-terminal part of Su(Hw) carries the domain (716–892 a.a.) that is responsible for insulator activity [32, 92, 95] and the ability of the Su(Hw) protein to inhibit transcription of the central nervous system (CNS) genes in the ovaries [96, 97, 98]. Two more proteins, Mod(mdg4)-67.2 and CP190, are recruited to the complex via direct interaction with Su(Hw) (*Fig. 3*).

The Mod(mdg4)-67.2 protein is produced by a complex locus, *mod(mdg4) *[99, 100]. At the N-end of the Mod(mdg4)-67.2 protein, there is the BTB/POZ domain (bric-à-brac, tramtrack and broad complex/ poxvirus and zinc finger), which widely occurs in higher eukaryotes and is usually homodimerized. However, the BTB domain of Mod(mdg4)-67.2 belongs to a special insect-specific group [101]. The BTB domains belonging to this group can form both homo- and heteromultimeric complexes [102]. The C-end of the Mod(mdg4)-67.2 protein carries a specific domain interacting with the C-terminal region of Su(Hw) (716– 892 a.a.) [83, 103]. Furthermore, the N-terminal part of the Su(Hw) protein interacts with the glutamine-rich region of the Mod(mdg4)-67.2 protein [104] (*Fig. 3*). The Mod(mdg4)-67.2 protein is involved in the enhancer-blocking activity of the Su(Hw) insulator.

The CP190 protein simultaneously interacts with Su(Hw) and Mod(mdg4)-67.2, thus stabilizing the formation of the insulator complex. The N-end of CP190 carries the BTB domain that forms stable homodimers [102, 105, 106, 107]. The C-end of CP190 carries glutamine-and asparagine-rich domains; between them, there reside the M domain responsible for interaction with microtubules and four ZFs [108]. The CP190 BTB domain interacts with two unstructured N-terminal regions of the Su(Hw) protein located between 88 and 202 a.a. [109]. The M domain of protein CP190 simultaneously interacts with the BTB domain of the Mod(mdg4)-67.2 protein [104, 110] (*Fig. 3*).

Deletions of separate domains in the Su(Hw), Mod(mdg4)-67.2, and CP190 proteins do not affect the *in vivo *assembly of the functional complex. Therefore, the Su(Hw) insulator forms through numerous interactions between its protein components, which partially compensate for and stabilize each other. Genome-wide studies have demonstrated that the complex containing all three proteins, CP190/Mod(mdg4)-67.2/Su(Hw), is assembled only at some Su(Hw)-binding sites [48, 94, 111]. The binding of the insulator complex to these sites is largely mediated by the CP190 and Mod(mdg4)-67.2 proteins [104, 109].

A new partner of Su(Hw), the HIPP1 protein (HP1 and insulator partner protein 1), has recently been identified [112]. Highly structured regions (1–212 and 675–778 a.a., respectively) reside at the ends of the HIPP1 protein; the C-terminal region corresponds to the crotonase domain [113, 114]. The crotonase domain of HIPP1 binds to the C-terminal region of Su(Hw) (637–892 a.a.), which is simultaneously responsible for the enhancer-blocking and repressive activities of the insulator. The N-terminal domain of HIPP1 interacts with the domains M and ZF of the CP190 protein [115] (*Fig. 3).* Inactivation of the *Hipp1 *gene was shown to affect neither the fertility of flies nor the Su(Hw)-dependent insulator activity [115, 116]. However, the simultaneous inactivation of the *Hipp1 *and *mod(mdg4)-67.2 *genes significantly changes the activity of the *gypsy *insulator and substantially weakens CP190 binding to Su(Hw)-dependent sites [115]. Therefore, the processes of HIPP1 and CP190 recruitment to the Su(Hw) insulator are mutually dependent.

It was also found that the ENY2 protein directly interacts with ZF10–12 of the Su(Hw) protein [117]. It was demonstrated for transgenic lines that the ENY2 protein is involved in the barrier activity of the Su(Hw) insulator and protects reporter gene expression against the PRE-dependent repression. Interestingly, ENY2 also binds to ZF of the dCTCF protein (CTCF ortholog in *Drosophila melanogaster*) and is involved in the barrier function of dCTCF-dependent insulators [118]. Recruitment of an unknown ENY2-dependent complex to the ZFs of various transcription factors (TFs) can potentially be regarded as the general mechanism of gene protection against PRE-dependent repression.

The RNA-binding proteins Shep and Rump, which act as negative regulators of enhancer-blocking activity, may be involved in the function of the Su(Hw)-dependent complex [119, 120]. Moreover, the activity of the Su(Hw) insulator can be regulated by the components of the RNA interference system: Ago, aub, piwi, and Rm62 [121]. However, the mechanism by which these proteins function has not been elucidated yet.

**Fig. 4:**
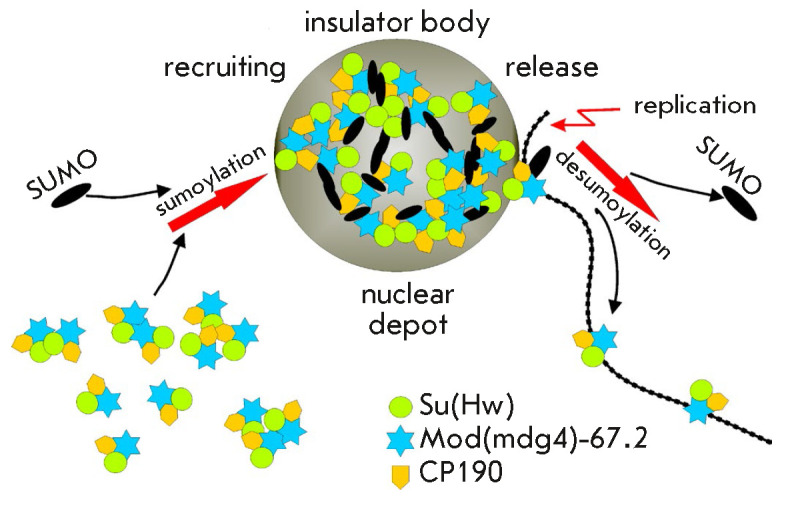
The model of formation and functioning of insulator bodies. Proteins CP190/Su(Hw)/Mod(mdg4)-67.2 are recruited into insulator bodies by sumoylation. In insulator bodies, Su(Hw)-dependent complexes are pre-assembled and associated with other TFs. The "matured" insulator complex transiently interacts with chromatin fibril, leaves the insulator bodies due to desumoylation, and binds to specific chromatin sites

Within the nucleus, the Su(Hw), CP190, and Mod(mdg4)-67.2 proteins reside in insulator bodies [122, 123]. Post-translational modification of the CP190 and Mod(mdg4)-67.2 proteins with a small ubiquitin-like modifier (SUMO) is needed to incorporate Su(Hw)-dependent proteins into the insulator bodies [122, 123, 124]. The dCTCF protein was also revealed within insulator bodies [125]. It was shown using *in vivo *model systems that formation of insulator bodies is unrelated to insulator activity [122], while sumoylation is not a necessary condition for the manifestation of enhancer-blocking activity [123]. It can be assumed that insulator bodies act as certain “depots” for chromatin proteins. Protein complexes, which efficiently bind to DNA synthesized during replication, are pre-assembled in these depots (*Fig. 4*).

The formation of insulator bodies is regulated by the amount of matrix protein EAST [124]. Under physiological conditions, the EAST protein does not bind to chromatin [126] but interacts with the CP190 and Mod(mdg4)-67.2 proteins [124]. The EAST expression level affects binding of the Su(Hw)-dependent complex to chromatin and the activity of Su(Hw)-dependent insulators [124, 127]. These effects of EAST can be interpreted using the model described above, according to which the insulator complexes are pre-assembled in insulator bodies.

## BRIEF CHARACTERIZATION OF INSULATOR PROTEINS

Most insulator complexes form around one or several key DNA-binding proteins. There are no clearly defined parameters according to which a protein can refer to insulator proteins. Therefore, any protein found within one or several insulators is automatically classified as belonging to the group of insulator proteins. *D. melanogaster *is known to have 11 proteins exhibiting enhancer-blocking properties that contain DNA-binding domains. Many of them (dCTCF, Su(Hw), Pita, ZIPIC, and GAF) contain C2H2-ZFs [128, 129, 130]. So far, only one conserved insulator protein, CTCF, has been described in vertebrates [131].

The CTCF protein is expressed in most mammalian tissues [132]. It is required during the early stages of mouse development and is involved in the cell cycle, apoptosis, and cell differentiation [133, 134, 135]. A CTCF ortholog having a similar domain structure (dCTCF) was found in Drosophila [136]. The dCTCF protein binds to most boundaries in the BX-C and is responsible for their insular activity. The central part of CTCF in vertebrates and Drosophila contains a cluster carrying 11 ZFs. The studies focused on the human CTCF–DNA complex have shown that ZFs 3–7 bind to the 15-bp consensus motif [137]. It was demonstrated using mutations in individual ZFs that in primary murine lymphocytes, ZFs 9–11 and ZFs 1–2 bind to the sequences flanking the consensus motif, thus stabilizing specific CTCF binding [138]. An unstructured domain forming homodimers resides at the N-end of CTCF in various organisms [139]. A motif interacting with the cohesin complex was also found at the N-end of human CTCF [140]. CTCF interacts with the cohesin complex to form chromatin loops and most of the TAD boundaries; it also mediates short-range interactions between the regulatory elements [90, 132, 141].

The ZIPIC, Pita, and Zw5 proteins carry the zinc finger-associated domain (ZAD) at their N-end and ZF clusters at their C-end [27, 68, 142, 143]. These proteins are intensively expressed at all stages of Drosophila development, especially during the embryonic stage. Mutations inactivating the *pita *and *zw5 *genes cause early embryonic death, thus indicating that the *Pita *and *Zw5 *proteins play an important role in gene expression regulation [27, 144]. The Zw5 protein was first detected on the *CG31211 *gene promoter, a part of the *scs *insulator [27]. An analysis of whole-genome distribution of the ZIPIC, Pita, and Zw5 proteins showed that they preferentially bind to gene promoters near transcription start sites and, like the CTCF protein, are often colocalized with components of the cohesin and condensin complexes [48, 145]. Thanks to the ZAD domains, the ZIPIC, Pita and Zw5 proteins can form homodimers [145]. In transgenic lines, the multiple binding sites of these proteins form insulators inhibiting the enhancer activity and PRE-dependent repression [146].

The GAF protein is involved in the functioning of the Fab-7 insulator from the BX-C [70], SF1 insulator from the ANT-C [52], and the insulator located between the *myoglianin *and *eyeless *genes [56]. A single ZF binding to the GAGAG motif resides in the central part of the protein [147, 148]. Similar to the Mod(mdg4)-67.2 protein, the N-end of GAF carries an insect-specific BTB domain that forms homo- and heteromultimers [101, 102]. The BTB domains GAF and Mod(mdg4)-67.2 can interact with proteins from different transcription complexes [102, 149, 150, 151].

The BEAF-32 protein was initially identified as a factor interacting with the *scs’ *insulator [30, 152]. To bind to DNA, BEAF-32 uses the N-terminal C2H2-like domain called BED. There is a BESS domain at the C-end of the protein, which is required for BEAF trimerization [152, 153]. Each subunit of the BEAF complex binds one CGATA motif, while BEAF trimers bind to clusters of the CGATA motif with high affinity [152]. Whole-genome analysis shows that BEAF is predominantly associated with the promoter regions of active genes and is involved in transcription stimulation [154, 155].

Identically to BEAF-32, the Ibf1 and Ibf2 proteins (insulator binding factors 1 and 2) bind to DNA through the BED domain to form hetero-oligomers [156]. A whole-genome analysis showed that Ibf1/Ibf2 is often simultaneously present with other insulator proteins, primarily with CP190 and dCTCF.

Elba1 and Elba2, the components of the recently discovered Elba (Early boundary activity) insulator complex, use conserved C-terminal BEN domains to bind to DNA [57]. The third protein, Elba3, is responsible for the formation of the Elba1/Elba2 dimer, which interacts with specific insulator sites. The Elba2 protein is expressed at most developmental stages, but two other components of the complex are present only during the early stage of embryonic development. Elba recognizes the 8-bp asymmetric CCAATAAG sequence, which is a part of the Fab-7 insulator from the BX-C. Another protein, Insv (Insensitive), binds to the Fab-7 insulator [157, 158]. Similar to the Elba protein, this protein carries the C-terminal BEN domain and is preferentially expressed in early embryos [158]. The Elba complex and Insv protein are needed to ensure *in vivo *functioning of the Fab-7 insulator [57, 157].

All the afore-listed insulator proteins found in Drosophila (except for Zw5 and the Elba complex) interact with the CP190 protein [68, 105, 108, 125, 156, 158-162]. DNA-binding insulator proteins recruit CP190 to chromatin [68, 105, 108, 161]. Meanwhile, the CP190 protein binds to most housekeeping gene promoters [108, 159, 161] and is involved in open chromatin formation [163]. The presence of the CP190 protein on insulators and promoters indicates that a functional relationship between them is possible.

## DIRECT PARTICIPATION OF INSULATORS IN ENHANCER-PROMOTOR INTERACTIONS

Most binding sites of insulator proteins were detected in the promoter regions of different genes [47, 48]. It is known that generation of active promoters is one of the key functions of mammalian CTCF protein [164]. The involvement of the same proteins in the formation of promoter and insulator complexes agrees with the transcription models of insulator action.

In transgenic Drosophila lines, the *gypsy *insulator completely blocks the *yellow *gene enhancers, which are isolated by it from the promoter, while having no effect on basic promoter activity [7]. However, if the *yellow *gene promoter is weakened by a mutation, the *gypsy *and 1A2 insulators restore its activity regardless of their positions in the transgene [165]. Like active promoters, Su(Hw)-dependent insulators recruit the SAGA and Brahma complexes formed on the regulatory elements of the open chromatin domain [166]. Su(Hw) insulators potentially compensate for the partial inactivation of the *yellow *promoter by recruiting remodulating complexes to it. Therefore, the insulator-bound complexes are supposed to reside in close proximity to the promoter. Indeed, it has been shown that in transgenic lines, insulators facilitate long-range interactions between the promoters and GAL4 activators residing at the 3’-end of the reporter genes [165, 167]. ChIP and 3C assays revealed an interaction between an enhancer located upstream of the *white *gene promoter and the *gypsy *insulator at the 3’-end of the gene [168]. Short-range interactions between regulatory elements are probably ensured by the proteins binding simultaneously to insulators and promoters [47, 48, 160, 169]. It was shown that the CP190, Chromator, and BEAF-32 proteins can ensure long-range interactions between chromatin domains [107]. It is fair to assume that the main function of the endogenous insulators residing at the 3’-end of the *yellow *and *white *genes [45, 46, 55] is to enhance the activity of the promoters of these genes.

All other insulators exhibit a much weaker blocking activity against *yellow *gene enhancers compared to the *gypsy *insulator [55, 64, 68, 170]. On the other hand, the *gypsy *insulator integrated into the transgenes between the enhancer and the *white *gene promoter only slightly weakens the *white *gene expression in fruit fly eyes [168]. Interestingly, the C-terminal domain of the Su(Hw) protein is simultaneously responsible for the blocking of the *yellow *gene enhancers and repression of the promoters of the CNS genes in female gonads [171]. The Su(Hw) binding sites are located directly in the promoters of the CNS genes [98]. It is most likely that repression occurs due to the recruitment of a repressor complex specific to the germinal tissue, since no repression is observed in the eyes [28].

In the absence of the Mod(mdg4)-67.2 protein, the *gypsy *insulator becomes a repressor of the *yellow *gene promoter [83, 95, 110]. It is noteworthy that the Mod(mdg4)-67.2 protein is recruited to the insulator complex through the C-terminal domain of Su(Hw) being responsible for insulation/repression. Repression in the *yellow *locus can be attributed to the fact that the efficiency of binding between the repressor complex and the C-terminal domain of Su(Hw) increases in the absence of the Mod(mdg4)-67.2 protein. It was shown that *gypsy*-dependent repression is mediated by the promoter sequence of the *yellow *gene, same as the sequence required for ensuring long-range enhancer-promoter interactions [172]. The Su(Hw)-dependent repressor complex potentially interacts with the promoter TF, thus ensuring communication with enhancers.

The reported experimental data confirm the model according to which insulators dynamically interact with enhancers and promoters. When an insulator is integrated between an enhancer and a promoter, the interaction between the insulator complex and TF of the promoter or the enhancer prevents efficient interaction between them. Thus, it was shown that the Mod(mdg4)-67.2 protein interacts with the Zeste protein. The Zeste protein binds to the *white *gene enhancer and promoter, thus providing communication between them [173, 174]. The interaction between the Mod(mdg4)-67 and Zeste proteins may interfere with the proper formation of enhancer-promoter contacts and reduce transcription. If the insulator recruits repressor complexes to the promoter region, enhancer activity is completely blocked.

Vertebrate CTCF protein often forms chromatin loops by interacting with active promoters [175, 176]. CTCF directly interacts with TAF3 and TFII-I, the components of the TFIID promoter complex [177, 178]. Therefore, CTCF-promoter interactions can prevent the formation of enhancer-promoter contacts. In the mammalian *Igf2/H19 *locus, the genes are located so as to ensure that the *H19 *gene in the maternal allele and the *Igf2 *gene in the paternal allele are activated by common distal enhancers [75]. The *H19 *gene is activated in the maternal allele, and the *Igf2 *gene is activated in the paternal allele. The interaction between the common enhancers and gene promoters is regulated by a CTCF-dependent insulator residing in the imprinting control region (ICR). A 3C assay showed that in the maternal allele, the CTCF protein ensures direct interaction between the insulator and *Igf2 *promoter, which inhibits the activation of *Igf2 *by distal enhancers [179, 180, 181]. Interestingly, the CTCF protein recruits the polycomb repressive complex 2 (PRC2) repressing transcription to the *Igf2 *promoter [181].

## THE ROLE OF CHROMATIN LOOPS IN ENHANCER BLOCKING

The structural models of insulator action postulate that chromatin loops and TADs block interactions between the regulatory elements from adjacent domains [85, 182, 183]. However, the ability of chromatin loops to completely block the enhancer-promotor interactions has not been verified experimentally.

**Fig. 5:**
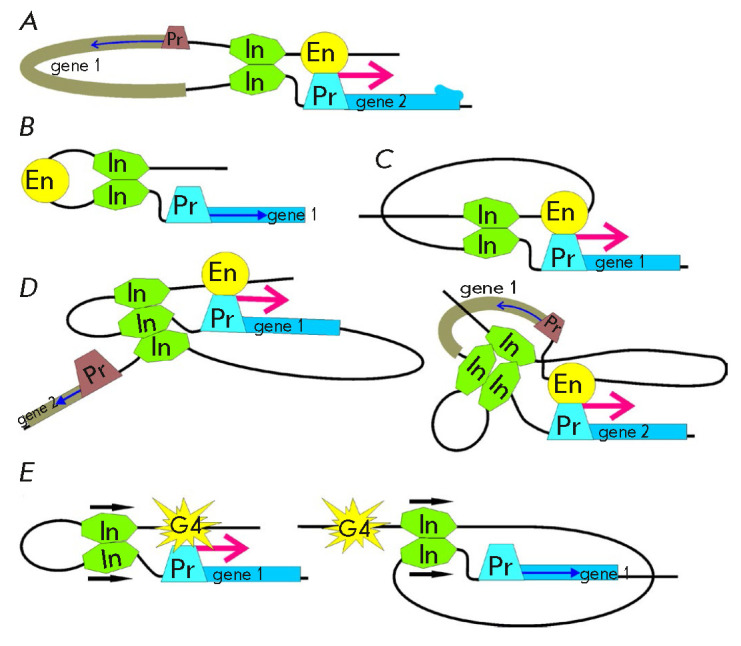
Modeling chromatin loops in transgenic lines of drosophila. (*A*) A loop formed by identical insulators brings the enhancer closer to the promoter. (*B*) A tight loop between the two insulators blocks the enhancer it contains. (*C*) Increased distance between the insulators surrounding the enhancer neutralizes the insulation. (*D*) Loops formed by the three insulators do not interfere with activation of the reporter gene transcription. (*E*) Mutual orientation of insulators (indicated with arrows) determines the configuration of the chromatin loop and, as a consequence, the possibility of transcription activation. Designations: G4 – yeast activator GAL4; other designations are the same as those in *Fig. 2*.

The functional role of the chromatin loops formed by insulators was thoroughly studied in transgenic Drosophila lines. It was found that two identical insulators integrated between the enhancer and the promoter mutually neutralize each other’s activities [55, 170, 184, 185, 186]. To interpret this phenomenon, it was suggested that the same insulators interact with each other more efficiently than with an enhancer or a promoter. Therefore, they do not interfere with the enhancer-promoter interactions and even facilitate the long-range communication between the regulatory elements. This model was confirmed by the experiments where another gene surrounded by insulators was located between the enhancer and the promoter of the reporter gene [59, 186, 187, 188]. Efficient enhancer-dependent activation of the reporter gene was observed only in the presence of insulators. Therefore, the chromatin loop formed by a pair of identical insulators brought the enhancer and the promoter closer together (*Fig. 5A*). Similar results were obtained using the lines in which the enhancer was replaced with the transcription-repressing PRE [189]. The gene residing between two *gypsy *insulators was protected against PRE-dependent repression. Meanwhile, the interaction between the insulators brought PRE closer to the second gene, thus leading to its repression. The physical interaction between insulators and the approximation of PRE to the second reporter gene was confirmed by 3C assay [190].

Mutual neutralization of two identical insulators makes it possible to study the direct role played by the chromatin loop formed by them in the blocking of the enhancer-promoter contacts. As mentioned above, the integration of a single copy of the *gypsy *insulator between the enhancer and the *white *gene promoter weakens the enhancer activity only slightly [168]. However, surrounding the enhancer with a pair of *gypsy *insulators completely inactivates it. This result suggests that the formation of a small chromatin loop containing an enhancer either topologically or sterically prevents productive interaction between the enhancer and the *white *gene promoter (*Fig. 5B*). Meanwhile, in transgenic lines, a single copy of the *gypsy *insulator completely blocks the enhancers activating *yellow *gene expression in the body and wings [170, 191]. It turned out that integration of the second copy of the *gypsy *insulator upstream of the enhancers (~ 8 kbp upstream of the first enhancer) restores *yellow *gene expression. Thus, formation of the 8-bp chromatin loop neutralizes the insulator activity (*Fig. 5C*). The insulator activity was completely restored when the distance between the surrounding *yellow *insulators was decreased to 2 kbp. Therefore, only small chromatin loops containing the enhancer can completely block its activity. *In vivo*, chromatin loops are much larger than 2–3 kbp, suggesting that interactions can exist between the regulatory elements residing in neighboring loops or loops located at a distance.

Studies performed for Drosophila lines carrying three copies of Su(Hw) insulators integrated between the enhancers and two reporter genes in different combinations showed that all three copies interact with each other [170, 191]. The chromatin loop formed around the enhancer or the reporter gene did not induce insulator activity. This result confirms once again that chromatin loops do not play a crucial role in the blocking of enhancer–promoter interactions (*Fig. 5D*).

In transgenic Drosophila lines, pairs of some insulators (e.g., *gypsy*, *Mcp*, and *Fab-7*), can be involved in ultra-long-range interactions (at a distance as large as several hundred thousands of nucleotide pairs) [192, 193]. The *Homie *and *Nhomie *insulators were detected at the boundaries of the *eve *locus expressing *pair-rule *TF that is involved in embryonic development [194]. These insulators efficiently interact with each other in transgenic Drosophila lines and can maintain ultra-long-range interactions between enhancers and the promoter of the *eve *locus in the genome [194, 195].

A model has been proposed to explain the mechanism of ultra-long-range interactions between insulators [16]. According to this model, insulators consist of binding sites for several proteins; each of those can be efficiently homodimerized. Indeed, the boundary of *Mcp *from the BX-C contains binding sites for Pita, dCTCF, and two other unknown insulator proteins [143, 196]. The *Fab-7 *boundary includes binding sites for GAF, Pita, Insv, Elba, the LBC complex, and several unknown proteins [57, 143, 157, 197, 198]. In transgenic Drosophila lines, paired binding sites for the Pita, ZIPIC, Zw5, dCTCF, and Su(Hw) proteins ensure long-range interactions between the reporter gene and yeast activator GAL4 [145, 146, 193]. However, any combination of the binding sites of different proteins results in a loss of interaction between insulators, thus confirming the contribution of protein homodimerization to long-range interactions.

Furthermore, the topology of chromatin loops depends on the mutual orientation of two identical insulators. This was demonstrated for the transgenic lines where GAL4 could not activate transcription of the *white *gene located at a long distance from it [146]. The identical insulators placed in close proximity to GAL4 and *white *promoter formed loops with two different configurations (*Fig. 5E*). If the insulators were oriented oppositely, GAL4 activated the *white *gene promoter. If the insulators had the same orientation, the resulting loop fully isolated GAL4 from the promoter. Similar results were obtained when the GAL4 activator was replaced with an enhancer [28, 187]. The mutual orientation of two *gypsy *insulators also affected the Flp-dependent recombination between FRT sites [199]. Oppositely oriented insulators located between the FRT sites contributed to recombination, whereas co-directional insulators inhibited it. Most likely, homodimerization of several proteins bound to identical insulators determines the direction of the interaction between them. The topology of the resulting chromatin loop regulates the interactions between the elements residing in close proximity to the insulators.

## MODERN VIEWS ON CHROMOSOMAL ORGANIZATION INTO TOPOLOGICALLY ASSOCIATING DOMAINS

**Fig. 6:**
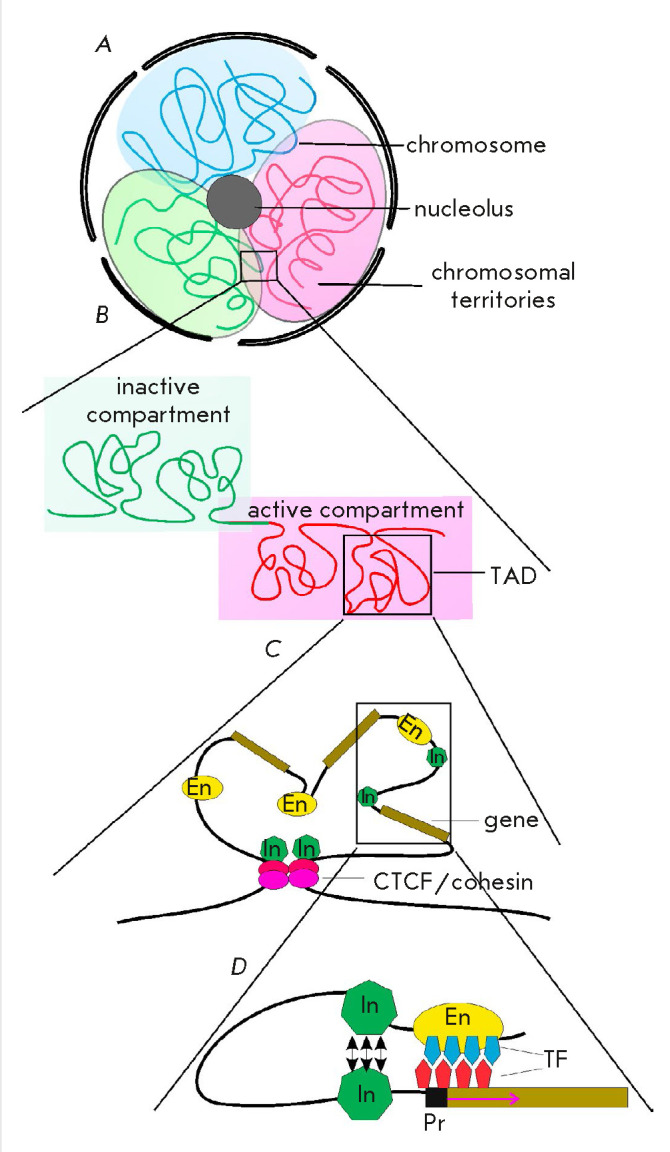
The levels of chromatin organization in the nucleus: (*A*) Chromosomes within the nucleus occupy particular territories (red, green, and blue backgrounds). (*B*) Each chromosome forms TADs, which are involved in a particular nucleus compartment depending on the active/ inactive chromatin state. (*C*) TADs facilitate the convergence of the regulatory elements within them and ensure synchronous gene expression. Architectural proteins can dynamically restrict the formation of TADs. (*D*) Insulators within a TAD may form local chromatin loops facilitating specific enhancer–promoter interactions. Designations: TFs – transcription factors; other designations are the same as those in *Fig. 2*

In all higher eukaryotes, chromosomes are organized into TADs. The size and mechanisms of formation of these domains greatly vary in different animal species [91, 200, 201]. Formation of TADs depends on the frequency of interaction between different chromatin parts: the interaction frequency within the domains is higher than that between the domains. Insulators inside TADs can form local chromatin loops, thus regulating the enhancer–promotion interactions (*Fig. 6*).

The CTCF protein and the cohesin complex interacting with it play the central role in the organization of TADs in mammals. Together with the cohesin complex, the CTCF protein resides at ~ 90% of TAD boundaries [89, 90]. The cohesin complex consisting of four subunits (SMC1, SMC3 and RAD21, SCC1) forms a ring-like structure around two DNA molecules [202]. It is believed that the cohesin complex can cause chromatin looping as chromatin passes through its ring-like structure (*Fig. 8A*). The cohesin complex slides along chromatin and forms loops; the binding sites of protein CTCF inverted with respect to each other act as limits for these loops [203, 204, 205]. Inactivation of CTCF or the cohesin complex components destroys most TADs, which agrees with the earlier described model [164, 206, 207]. The weak link in this model is the lack of experimental data that would confirm that the cohesin complex can cause chromatin looping *in vivo *[208].

**Fig. 7:**
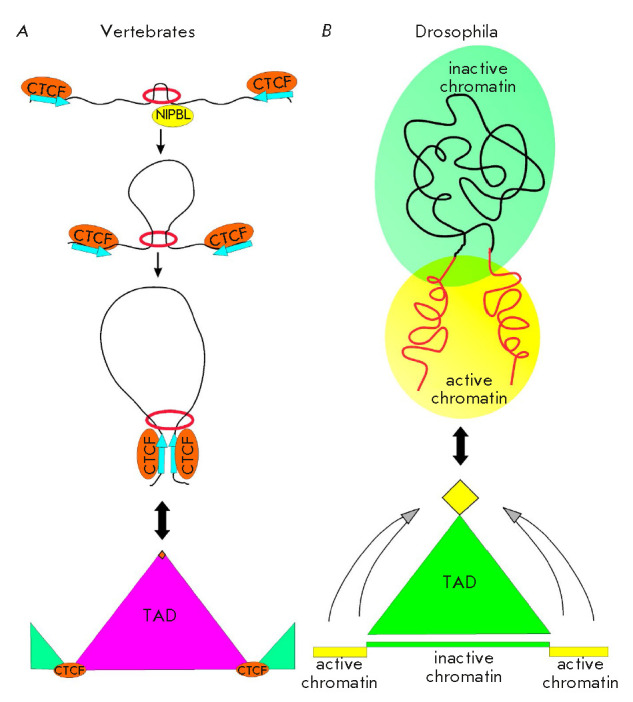
The mechanism of formation of TADs in vertebrates and drosophila. (*A*) Loop formation by the cohesin complex. The cohesin complex (red ring), after being loaded onto chromatin by NIPBL, processively extrudes chromatin through its ring-shaped structure, resulting in a growing chromatin loop. Loop extrusion stops when cohesin encounters CTCF binding sites in a convergent orientation (designated by arrows). Triangles represent the neighboring TADs divided with CTCF sites. An orange rhombus at the top of the TAD designates the high frequency of interaction between CTCF-binding regions. *(B) *In drosophila, active and inactive chromatin is localized in different nuclear compartments. Inactive chromatin (a green rectangle) is confined to the areas with active transcription (yellow rectangles). The interaction of actively transcribed regions (shown with arrows) forms TAD. The yellow rhombus at the top of the TAD denotes the highest frequency of interaction between active chromatin regions

In mammals, the role of CTCF-binding sites in the formation of TAD boundaries was studied in the murine *Hox *genes [209]. The *HoxA *and *HoxC *genes are located in the adjacent TADs and are transcribed independently. Deletion of the CTCF-binding site residing between these TADs destroyed their boundaries, thus altering the gene expression patterns and, therefore, causing homeotic transformation of the skeleton [210]. Unlike *HoxA *and *HoxC*, the *HoxD *gene is located between two TADs, each containing enhancers responsible for the function of *HoxD *in a certain tissue type. In this case, however, deletion of CTCF-binding sites in the *HoxD *gene did not destroy the TAD boundary and had a minimal impact on the gene expression pattern. The TAD boundary was destroyed, and the pattern of *HoxD *expression changed only after an extensive deletion affecting the structure of the regulatory regions of the gene. These data indicate that some additional TFs, along with CTCF and the cohesin complex, can be involved in the formation of TAD boundaries.

Unlike in vertebrates, dCTCF and the cohesin complex in Drosophila are not the key factors in TAD formation. The TADs being formed correlate well with epigenetic marks and are subdivided into classes corresponding to the specific features of chromatin: (1) the active TADs are actively transcribed and are rich in H3K4me3 and H3K36me3 histone modifications; (2) the polycomb-dependent TADs are rich in H3K27me3 histone modification and Polycomb group proteins; (3) “null” or “void” TADs have no known specific histone marks; and (4) heterochromatic TADs are rich in H3K9me2 mark and the HP1 and Su(var)3-9 proteins [91]. Chromatin regions separating the TADs are rich in genes with a high transcription level [211, 212, 213]. They actively interact with each other to form chromatin loops. There are no clearly defined sites of TAD formation such as inverted CTCF sites in mammals [211].

**Fig. 8:**
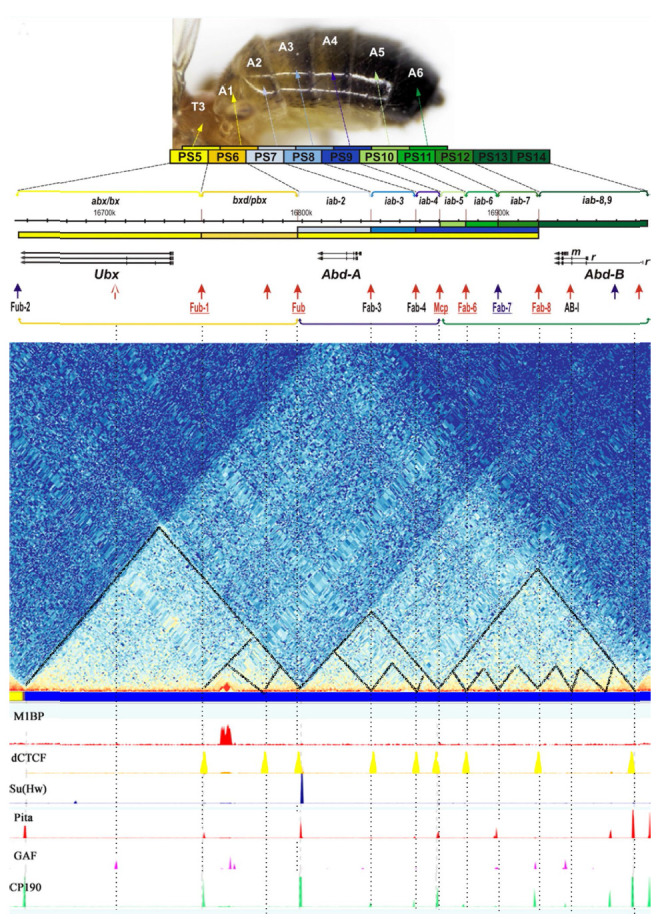
Schematic representation of BX-C. The BX-C map and coordinates are taken from the FlyBase resource (R6.04). The colored rectangles represent the embryonic parasegments (PS) corresponding to the imago segments. The regulatory regions controlling the expression of the *Ubx*, *abd-A*, and *Abd-B *genes (horizontal arrows) in each PS are indicated with upper brackets. The regulatory regions are organized into three transcriptionally associated regions indicated with lower brackets. The pattern and expression level of each gene are designated by colored scale; the darker color indicates higher expression levels. BX-C insulators are indicated with arrows: red arrows denote the CTCF-dependent ones; blue arrows denote the CTCF-independent ones [223]. The distribution map of TADs and some insulator/ architectural proteins in BX-C was constructed using the Chorogtnome Navigator dm3 resource [212]

Hence, the TAD boundaries in Drosophila are more likely to depend on the active chromatin state and its properties rather than on the binding sites of a specific protein [213] (*Fig. 8B*). The dCTCF, CP190, Chromator, Z4, and BEAF-32 insulator proteins binding to housekeeping gene promoters are often found at the TAD boundaries [201, 211, 212, 214]. However, the role played by these proteins in TAD boundary formation still needs to be elucidated.

Recent studies focused on chromatin architecture in individual mammalian cells have revealed the high heterogeneity of TAD boundary localization [215-218]. Meanwhile, DNA sites within the TADs interact on average only two to three times more frequently than sites from the adjacent TADs [89]. The transboundary interactions were confirmed by FISH [219, 220]. These results agree with the vigorous dynamics of binding/ dissociation of the CTCF protein, which resides on chromatin for approximately 2 min [221]. Therefore, TAD formation is a dynamic process and TAD boundaries are not a rigid barrier limiting the enhancer-promotor interactions.

## THE ROLE PLAYED BY INSULATORS AND TADS IN TRANSCRIPTION REGULATION

Drosophila insulators play a significant role in ensuring specific long-range *cis*-regulatory interactions, which has been demonstrated well for the BX-C [222]. The *Ubx, Abd-A*, and *Abd-B *homeotic genes within the BX-C are responsible for the formation of the third thoracic and all the abdominal segments of a fruit fly and determine its future head-to-abdomen axis. The BX-C is divided into nine regulatory domains (*iab 1–9*), each activating specific transcription of one out of three homeotic genes in a certain segment (*Fig. 8*). The BX-C contains two TADs whose shared boundary coincides with the *Fub *insulator residing between the regulatory domains of the *Ubx *and *Abd-A *genes [217] (*Fig. 8*). The *Mcp, Fab-6, Fab-7*, and *Fab-8 *insulators have been the best studied. They determine the boundaries of the *iab- 5*, *iab-6*, and *iab-7 *domains that regulate the *Abd-B *expression level in the A5, A6, and A7 abdominal segments [222, 223]. The entire regulatory domain of the *Abd-B *gene is located within a single TAD. In the A5 segment, *iab-5 *enhancers are active, while *iab-6 *and *iab-7 *enhancers are inactive. The *iab-6 *enhancers ensuring stronger activation of *Abd-B *expression are active in the next segment (A6). Even stronger *iab-7 *enhancers are active in the A7 segment. Therefore, *Abd-B *expression is enhanced in every segment thereafter, which is responsible for proper development of each abdominal segment. The interactions between the adjacent regulatory domains are blocked by insulators. For example, premature activity of *iab-6 *enhancers in the A5 segment is observed when the *Fab-6 *insulator is deleted.

*In vivo *genome editing made it possible to thoroughly study the structure and functions of insulators at the BX-C boundaries. It turned out that insulators consist of two modules: one blocking the communication between the adjacent regulatory domains (the insulator module) and the other one ensuring specific interaction between the insulator and the promoter of the *Abd-B *gene (the communicator module) [224, 225]. The Su(Hw), Pita, and dCTCF proteins, as well as the CP190 protein interacting with them, are involved in local insulation of the regulatory elements residing in the neighboring domains [143, 196, 226] (*Fig. 8*). The insulator module may consist of any combination of binding sites for these proteins, but there must be at least four sites. The communicator module of all insulators carries the binding sites of the poorly studied LBC complex comprising the GAF and CLAMP proteins [198, 224]. The communicator modules interact with the pre-promotor domain of the *Abd-B *gene to form chromatin loops ensuring specific contacts between *iab *enhancers and the *Abd-B *promoter. SubTADs corresponding to individual *iab *domains have been revealed in embryonic cell populations [217]. The formation of subTADs correlates with activation of *iab *domains, being potentially indicative of interaction between active domains and the *Abd-B *promoter. This fact confirms that TADs are formed in *D. melanogaster *through the interaction between active chromatin sites, while insulator proteins stabilize the boundaries of the resulting domains.

**Fig. 9:**
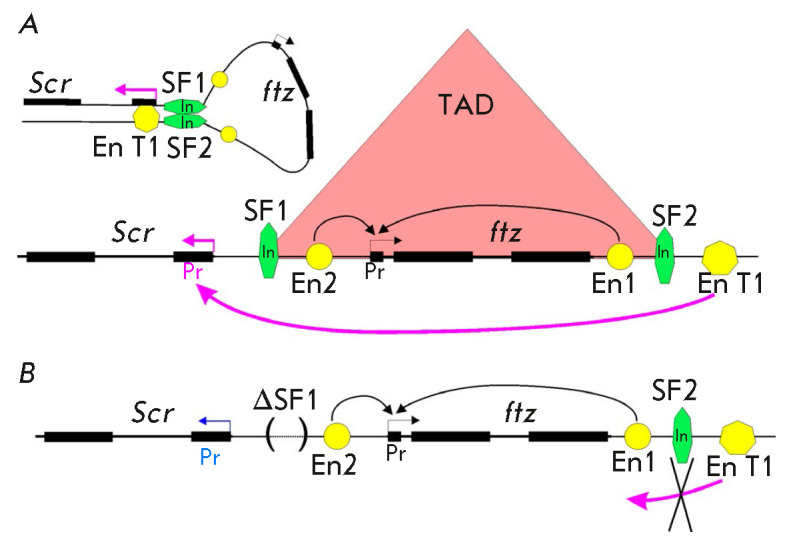
The role played by the SF1 and SF2 insulators in TAD formation and transcription in ANT-C. (*A*) The boundaries of the TAD including the *ftz *gene are determined by the SF1 and SF2 insulators. Interacting insulators form a loop that brings the T1 enhancer closer to the *Scr *gene promoter. The T1 enhancer activates *Scr *transcription. (*B*) Deletion of the SF1 insulator (designated by brackets) leads to disruption of TAD but not misexpression of the *ftz *gene. However, the T1 enhancer does not activate *Scr *transcription, because a loop between insulators does not form. All designations are the same as those in *Fig. 2*

The formation/destruction of TADs can only have a minimal effect on gene expression [164, 206, 227]. Thus, the TAD boundaries in the complex of homeotic ANT-C genes are determined by two insulators: SF1 and SF2 [53, 228] (*Fig. 9A*). Deletion of the SF1 insulator results in TAD destruction, while having no effect on the expression of the *fushi-tarazu *(*ftz*) gene residing inside the TAD. Interestingly, transcription of the *Scr *gene adjacent to the TAD is reduced [229] (*Fig. 9B*). In early embryos, the *Scr *gene located on one side of the TAD is activated by directly interacting with the T1 enhancer residing on the other side of the TAD [230] (*Fig. 9A*). Therefore, the interacting SF1 and SF2 insulators on the TAD boundaries bring together the T1 enhancer and the *Scr *gene. This situation fully implements the model developed for transgenic lines, according to which chromatin loop formation between insulators located at a distance contributes to enhancer-promotion interactions and transcription activation.

**Fig. 10:**
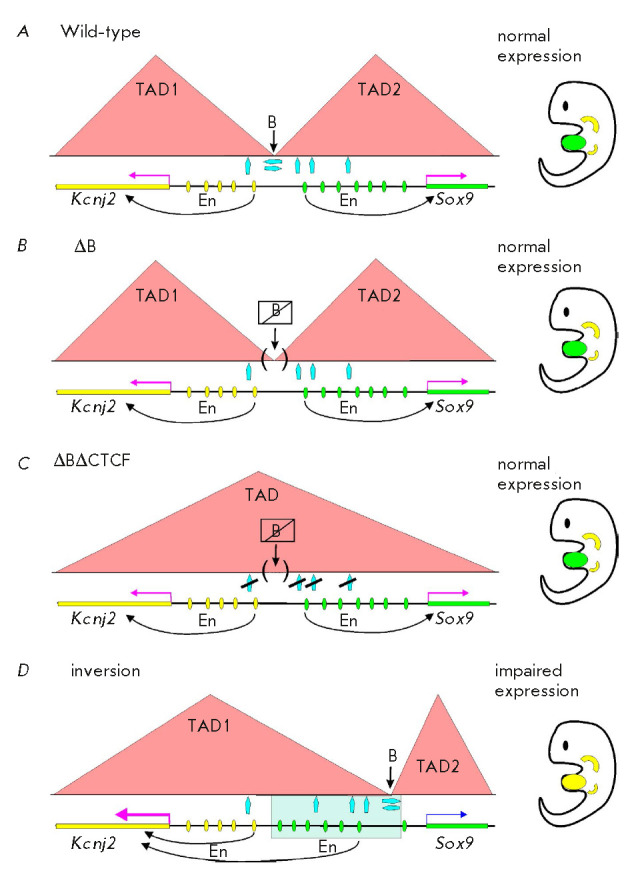
The role of TADs in *Kcnj2 *and *Sox9 *loci expression. *(A) *Wild-type expression of the *Kcnj2 *and *Sox9 *genes. Two separate TADs are formed, the boundary (B) between which it colocalizes with the convergently oriented CTCF binding sites. (*B*) Deletion of CTCF-binding sites at the TAD boundary neither destroys them nor affects the gene expression patterns. (*C*) Simultaneous deletion of boundary and internal CTCF sites leads to fusion of TADs but does not affect gene expression. (*D*) Relocation of the boundary between TADs results in gene misexpression. Designations: blue arrows – CTCF-binding sites; the expression patterns of the *Kcnj2 *and *Sox9 *genes in the embryo are shown in yellow and green, respectively; the direction of enhancer action is shown with arrows; other designations are the same as those in *Fig. 2* and *Fig. 9*.

Furthermore, the effect of TAD boundaries on transcription was studied by performing precise deletion of different CTCF-binding sites in the *Sox9–Kcnj2 *locus in mice [231]. Two TADs separated by a boundary containing inverted CTCF-binding sites resided in this locus (*Fig. 10A*). Several additional CTCF sites are also found inside each TAD. The *Sox9 *and *Kcnj2 *genes are activated by specific enhancers and have different expression patterns. Deletion of CTCF-binding sites on the boundary between the *Sox9 *and *Kcnj2 *genes did not cause merging of the TADs (*Fig. 10B*). A merged TAD was formed only after additional internal CTCF sites had been deleted (*Fig. 10C*). It is noteworthy that during TAD merging, the enhancers did not activate the nonspecific gene and expression of the *Sox9 *and *Kcnj2 *genes remained almost unchanged. It is possible that the high specificity of enhancer-promotor interactions did not allow the cohesion complex to form new contacts between the regulatory elements in the shared *Sox9*–*Kcnj2 *locus. Therefore, the TAD boundary was not involved in the organization of specific enhancer-promotor interactions. Inversion, which had moved the TAD boundary to a position between the *Sox9 *gene enhancers and its promoter, resulted in the formation of two new domains (*Fig. 10D*). In this case, the TAD boundary had a critical impact on transcription. The *Sox9 *enhancers isolated from the promoter could not activate the specific gene but activated *Kcnj2*, which had a lethal effect.

These examples allow one to infer that chromosomal organization into topological structures and specific enhancer-promotor interactions are two different transcription regulation levels that are often independent. Only in some cases do the TAD boundaries act as insulators regulating the enhancer-promoter interactions.

The correlation between gene expression and an altered chromatin architecture was also studied in Drosophila lines carrying chromosomes with multiple inversions and deletions [232]. It was revealed that significant changes in the TAD organization have a negligible effect on gene transcription. These data once again indicate that TADs play a secondary role in gene expression regulation in higher eukaryotes.

## CONCLUSIONS

Today, it is obvious that TADs form the chromosomal architecture but do not act as transcription domains regulating gene expression. In *Drosophila melanogaster*, most TAD boundaries are formed by promoters of actively transcribed genes. In some cases, the TAD boundaries coincide with insulators. Interestingly, many proteins binding to insulators are also components of the complexes assembled on promoters. Insulators are the multifunctional regulatory elements. They ensure the specificity of enhancer-promotion interactions, form the boundaries between active and inactive chromatin, and form the regions containing open chromatin available for TF. The experimental data demonstrate that insulators inhibit enhancer activity by directly interacting with enhancers or promoters. Chromatin loops formed by insulators play only an auxiliary role in insulation. The question of how long-range interactions between enhancers, silencers, promoters, and insulators form and are regulated still remains open. There is little doubt that insulator proteins play a crucial role in this process. However, their mechanism of action needs further study.

## Abbreviations

a.a.: amino acid
bp: base pair
kbp: kilobase pair
PRE: polycomb response element
LCR: locus control region
ANT-C: Antennapedia complex
TF: transcription factor
ZF: zinc finger
BX-C: Bithorax complex
CNS: central nervous system
BTB: Broad-complex, Tramtrack, and Bric-à-brac
POZ: poxvirus and zinc finger
ZAD: zink finger-associated domain
ICR: imprinting control region
PRC2: polycomb repressive complex 2
TAD: topologically associating domain
